# Virtual Reconstruction and Prey Size Preference in the Mid Cenozoic Thylacinid, *Nimbacinus dicksoni* (Thylacinidae, Marsupialia)

**DOI:** 10.1371/journal.pone.0093088

**Published:** 2014-04-09

**Authors:** Marie R. G. Attard, William C. H. Parr, Laura A. B. Wilson, Michael Archer, Suzanne J. Hand, Tracey L. Rogers, Stephen Wroe

**Affiliations:** 1 Evolution and Ecology Research Centre, School of Biological, Earth and Environmental Sciences, University of New South Wales, Sydney, New South Wales, Australia; 2 Function, Evolution and Anatomy Research laboratory, Zoology, School of Environmental and Rural Sciences, University of New England, New South Wales, Australia; 3 Evolution of Earth and Life Sciences Research Centre, School of Biological, Earth and Environmental Sciences, University of New South Wales, Sydney, New South Wales, Australia; Friedrich-Schiller-University Jena, Germany

## Abstract

Thylacinidae is an extinct family of Australian and New Guinean marsupial carnivores, comprizing 12 known species, the oldest of which are late Oligocene (∼24 Ma) in age. Except for the recently extinct thylacine (*Thylacinus cynocephalus*), most are known from fragmentary craniodental material only, limiting the scope of biomechanical and ecological studies. However, a particularly well-preserved skull of the fossil species *Nimbacinus dicksoni*, has been recovered from middle Miocene (∼16-11.6 Ma) deposits in the Riversleigh World Heritage Area, northwestern Queensland. Here, we ask whether *N. dicksoni* was more similar to its recently extinct relative or to several large living marsupials in a key aspect of feeding ecology, i.e., was *N. dicksoni* a relatively small or large prey specialist. To address this question we have digitally reconstructed its skull and applied three-dimensional Finite Element Analysis to compare its mechanical performance with that of three extant marsupial carnivores and *T. cynocephalus*. Under loadings adjusted for differences in size that simulated forces generated by both jaw closing musculature and struggling prey, we found that stress distributions and magnitudes in the skull of *N. dicksoni* were more similar to those of the living spotted-tailed quoll (*Dasyurus maculatus*) than to its recently extinct relative. Considering the Finite Element Analysis results and dental morphology, we predict that *N. dicksoni* likely occupied a broadly similar ecological niche to that of *D. maculatus*, and was likely capable of hunting vertebrate prey that may have exceeded its own body mass.

## Introduction

Thylacinids first appear in the Australian fossil record during the late Oligocene (∼24 Ma) and include the largest representatives of the Dasyuromorphia, i.e., families Thylacinidae, Dasyuridae and Myrmecobiidae [1]–[8]. A wide range of feeding ecologies are known within the order. They include omnivores, insectivores, small prey specialists, hypercarnivores and osteophageous species [9]. Variation in the dentition, skull shape and body size (∼1–60 kg) of thylacinids suggests considerable trophic diversity within the family [10], [11]. In addition to the recently extinct thylacine or Tasmanian ‘tiger’ (*Thylacinus cynocephalus*), eleven extinct species of thylacinid have been described [4], [12]–[18]. Up to five species may have co-existed in the Riversleigh World Heritage Area, northwestern Queensland between the late Oligocene (∼24 Ma) to middle Miocene (16-11.6 Ma) [12], [16]. See Table S1 for the temporal and geographic distribution of all thylacinid species.

The Riversleigh thylacinids inhabited forests [19], [20]. These regions were also occupied by an assortment of other carnivorous/omnivorous taxa, including ‘giant’ carnivorous rat-kangaroos (*Ekaltadeta* spp.), crocodiles (Mekosuchinae, e.g. *Baru darrowi* and *Trilophosuchus rackhami*), flightless dromornithid birds, marsupial lions (Thylacoleonidae), bandicoots (Peramelemorphia), dasyurids (Dasyuridae), pythons (Pythonidae), madtsoiid snakes and the world's oldest known venomous snakes [21]–[23]. Subsequent drying of the Australian continent from the late Miocene (11.6–5.3 Ma) led to the gradual replacement of forest environments with open woodlands, shrublands and grasslands [19]–[21]. These changes appear to broadly correlate with declining thylacinid diversity [24].

To date, interpretations of the ecology and feeding behavior of fossil thylacinids have been largely qualitative. This is, at least in part, because most extinct species are known only from jaw fragments and teeth. The near-complete skull of *Nimbacinus dicksoni* [4], [14], [18], a medium-sized thylacinid, provides an opportunity to more fully investigate feeding ecology in an extinct thylacinid.

*Nimbacinus dicksoni* was approximately 5 kg in body mass [10]. Fossils of *N. dicksoni* have been recovered from Oligocene-Miocene (∼24–5.3 Ma) deposits in the Riversleigh World Heritage Area, northwestern Queensland and Bullock Creek, Northern Territory [12], [14], [16], [18]. Its dentition is less specialized than that of the species of *Thylacinus*, but broadly similar to that of the living dasyurid, the spotted-tailed quoll (*Dasyurus maculatus*) in the arrangement and geometry of molar shearing crests typically associated with carnivory [18], [25].

To date conflicting evidence has been presented regarding the body size of prey *N. dicksoni* may have hunted. Predictions of bite force adjusted for body mass, based on application of 2D beam theory, have suggested that *N. dicksoni* may have taken relatively large prey, as does the slightly smaller *D. maculatus* [26]. However, shape analysis of the cranium has suggested that the species may have been restricted to smaller prey and/or included a higher proportion of invertebrate food in its diet [11].

The loads imposed on an animal during prey acquisition and feeding play an important role in the evolution of its skull morphology [27]. Testing hypotheses regarding the relationship between the form and function of skulls from extinct species requires an understanding of this relationship in living animals [28]. A comparative biomechanics approach involving living analogues has increasingly been applied to predict the feeding ecology and predatory behavior of extinct species [29]–[34]. To gain further insight in the feeding ecology of *N. dicksoni*, here we perform a biomechanical analysis of the skull of *N. dicksoni* to predict its mechanical behavior in response to loads simulating the capture and processing of prey.

Finite Element Analysis (FEA) is a computer modeling approach now commonly used by biologists and paleontologists to examine and compare mechanical performance in biological structures in comparative contexts [35]–[41]. In FEA, continuous structures, such as the skull, are divided into discrete, finite numbers of elements, allowing the prediction of mechanical behavior for complex geometric shapes. The structure is analyzed in the form of a matrix algebra problem that is solved with the aid of a computer [42].

Studies of feeding ecology for thylacinids have primarily focused on the most recently extinct member of the family, *T. cynocephalus*, which survived in Tasmania until 1936 [43]. Our understanding of the ecology of *T. cynocephalus* is chiefly based on morphological comparisons and 2D beam theory [11], [26], [44], [45], as well as anecdotal accounts of their behavior in the wild [46], [47]. Elbow joint morphology of *T. cynocephalus* evidently most closely resembles that of extant ambush predators, a compromise between efficient distance locomotion and the ability to manipulate and grapple with prey [48]. Three-dimensional biomechanical modeling of the skull of *T. cynocephalus*, extant dasyurids and an introduced Australian predator (*Canis lupus dingo*) have suggested potential limitations in prey body size [32], [35].

Morphological and biomechanical comparisons including sympatric native predators in Tasmania indicate that the diet of *T. cynocephalus* may have overlapped considerably with the two largest extant marsupial carnivores, the Tasmanian devil (*Sarcophilus harrisii*) and spotted-tailed quoll (*D. maculatus*) [35], [44], [49]. These species represented the three largest marsupial carnivores in Tasmania at the time of European settlement.

*Sarcophilus harrisii* is the largest living marsupial carnivore (mean adult male weight 8.7 kg, female 6.1 kg) [50]. They are the only specialized scavengers among living marsupials, filling a broadly similar ecological niche to that of osteophagous hyenas [44]. They are also opportunistic hunters and are known to prey on mammals that may exceed their body mass [51], [52]. Relative to its body size, its predicted bite force is greater than that of any other extant mammal studied to date [26].

Quolls are represented by four extant species in Australia and two in New Guinea [53]. The largest quoll, *Dasyurus maculatus* (maximum weight 7 kg) has a broad diet mainly consisting of mammals and insects, but will occasionally feed on birds and reptiles [54], [55]. Larger prey species constitute a higher proportion of the diet of adult male *D. maculatus*, while females and immature *D. maculatus* more frequently feed on smaller bodied mammals and invertebrates [56], [57]. The northern quoll (*Dasyurus hallucatus*) is the smallest and most arboreal of the four Australian quolls, weighing up to 1.2 kg [58], [59]. Although primarily insectivorous, this active hunter can feed on a variety of foods: fruits, small mammals, birds, reptiles, frogs and carrion [60]–[62].

In this study we aim to determine whether *N. dicksoni* was capable of killing large prey relative to their body size, or was restricted to catching relatively small bodied species. By digital reconstruction of its skull and applied 3D FEA, we compare its mechanical performance with that of three extant marsupial carnivores; *S. harrisii*, *D. maculatus* and *D. hallucatus*. We use previously applied scaling procedures [31] to account for differences in body mass, allowing for comparison of results between species. We predict that *N. dicksoni* will show similar distributions and magnitudes of craniomandibular stress to that of *D. maculatus* due to similarities in their dental morphology and size [18]. We also include *T. cynocephalus* to establish whether the biomechanical performance of *N. dicksoni* more closely resembles that of this larger, more derived thylacinid than dasyurids. A general assessment of the phylogenetic relationships of dasyuromophians, including taxa examined in this study is shown in Figure S1. We test if the biomechanical patterns and the inferred feeding aspects in *T. cynocephalus* in a recent study [35] are derived. We hypothesize that the relatively long rostrum of *T. cynocephalus* will result in higher stresses in the skull during biting and prey procurement than *N. dicksoni* and other dasyuromorphians.

## Materials and Methods

### Specimens

The skull of *Nimbacinus dicksoni* (QMF36357) was recovered from AL90 Site on the Gag Plateau of the Riversleigh World Heritage area in northwestern Queensland. Precise locality details can be provided on application to the Queensland Museum. This specimen was collected under permits issued by Queensland Department of Environment and Heritage and Environment Australia, and registered in the paleontological collections of the Queensland Museum, Brisbane, Australia. All necessary permits were obtained for the described study, which complied with all relevant regulations.

The biomechanical performance of the *Nimbacinus dicksoni* skull was compared with that of four dasyuromorphian species covering a range of craniodental morphologies and feeding ecologies. These comprized three extant dasyurids [*Dasyurus hallucatus* TMM M-6921; *D. maculatus* UNSW Z20; *Sarcophilus harrisii* AM10756] and one thylacinid (*Thylacinus cynocephalus* AM1821). Institutional abbreviations are QMF (Queensland Museum Fossil, Queensland), TMM (Texas Memorial Museum, Austin), UNSW (University of New South Wales, Sydney) and AM (Australian Museum, Sydney).

We generated 3D finite element models (FEMs) of each skull on the basis of computed tomography X-ray (CT) scan data. Digimorph (University of Texas; http://www.digimorph.org) was the source of CT data of a *D. hallucatus* skull (0.0784 mm slice thickness, 0.0784 mm inter-slice distance). Permission to use Digimorph derived CT scan data was granted by Dr. Timothy Rowe, Project Director of Digimorph. Other skulls were scanned in a Toshiba Aquillon 16 scanner (ToshibaMedical Systems Corporation, Otawara, Tachigi, Japan) at the Mater Hospital, Newcastle, NSW (1 mm slice thickness, 0.8 mm inter-slice distance, 240 mm field of view). The Australian Museum, Queensland Museum and University of New South Wales granted the loan of specimens to obtain CT data for this study.

We used the same specimen CT scans of *T. cynocephalus*, *D. maculatus* and *S. harrisii* as examined by Attard et al. [35], but constructed the FEMs again using a higher resolution mesh than that used by Attard et al. [35]. More specifically, the 3D surface meshes which formed the bases of the FEMs were computed using the High Quality as opposed to Medium Quality option in Mimics (ver. 13.2). The generated surface meshes were then converted to FEMs in STRAND7 (ver. 2.4) following previously established protocols [31], [35], [63]. Additionally, 3D objects of *T. cynocephalus* and all dasyurid skulls were exported as separate stl files for the creation of interactive 3D pdf documents that can be viewed using Adobe Reader (Figure S2, S3, S4, S5).

### Digital Reconstruction of *Nimbacinus dicksoni*

For detailed descriptions of *N. dicksoni* see Muirhead and Archer [14] and Wroe and Musser [18]. Previous analysis has suggested that, within the family, the dentition of *N. dicksoni* is less derived than the recent *Thylacinus cynocephalus* for at least 12 features, but that relatively few cranial specializations in *T. cynocephalus* distinguish the two species. These two taxa share at least three cranial features not present in the most generalized thylacinid known from significant cranial material, the late Oligocene *Badjcinus turnbulli* [14], [18].

**Figure 1:**
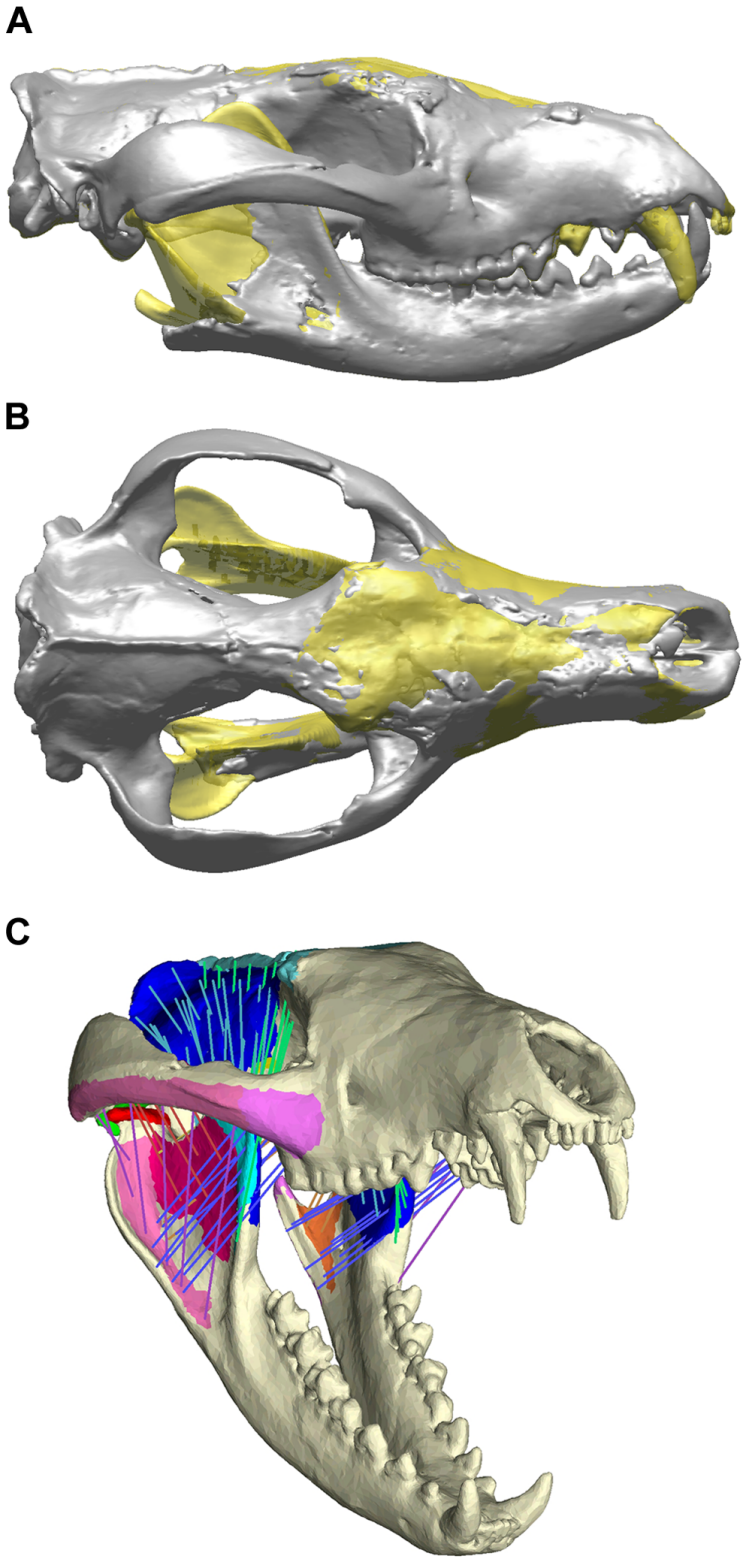
Digital reconstruction of *Nimbacinus dicksoni*. Original (grey) and reconstructed 3D (yellow) in (A) lateral view and (B) dorsal view. (C) Pre-processed Finite Element model of *N. dicksoni*, showing jaw musculature represented by trusses.

The skull of *N. dicksoni* is well preserved, although some regions are absent or damaged. Specifically, some damage/deformation is present at the postorbital processes, frontal, maxillary and nasal bones, which are compressed dorsoventrally (Figure 1). These damaged regions were reconstructed according to the morphology of surrounding bone regions once the damaged areas had been isolated and deleted [64]. Regions of bone that showed only minor damage were smoothed to create a coherent surface mesh for later solid meshing.

The right and left dentaries were largely intact but missing the superior regions of the coronoid processes, the temporomandibular joints (TMJ), condyles and angular processes (Figure 1). The anterior of the mandible is broken, separating both dentaries. We used the right dentary as a basis for reconstruction because its dentition was more complete, with only the incisors missing (Figure 1). We used a surface mesh of the right dentary of *D. maculatus* to reconstruct posterior regions of the right dentary of *N. dicksoni*. *Dasyurus maculatus* was chosen as its mandible was most similar in shape to that of *N. dicksoni* [11], thereby minimizing the extent of warping needed (and see below).

Reconstruction involved scaling the dentary of *D. maculatus* to the same size as that of *N. dicksoni* on the basis of skull length (condylo-basal). The missing posterior region of the *N. dicksoni* specimen was then isolated on the *D. maculatus* specimen and the mesh fitted to the existing structure in the mesh of *N. dicksoni* using Iterative Closest Point (ICP) registration. ICP is an algorithm that revises the transformation needed to minimize the distance between the points of two partially overlapping meshes. This process re-oriented the *D. maculatus* dentary in accordance with the morphology of the *N. dicksoni* dentary [65]. The anterior region of the *D. maculatus* dentary was deleted and the posterior region ‘warped’ so that overlapping regions of the coronoid process and angular process from the *D. maculatus* mesh matched the existing morphology of *N. dicksoni*. The manual warping method was used as much of the target (fossil) morphology was missing, making it impossible to apply homologous landmarks on both complete (*D. maculatus*) and incomplete fossil (*N. dicksoni*) specimens. Procedures to warp overlapping skull regions followed established protocols used by Oldfield et al. and Parr et al. [38], [66]. Manual warping works by establishing a grid of control points around the complete model (note that at this stage the incomplete fossil model has been ICP registered with the scaled complete model by matching the orientations of the regions of the jaw that are present in both models). These control points are then manipulated so that the surface morphology of the complete model matches that of the target (fossil). This is another variation of Template Mesh Deformation [66], but with the template points being the grid control points around the complete model rather than homologous anatomical points on both models.

Similarly, the TMJ of the complete (*D. maculatus*) model was warped so that the condyle articulated with and fitted the *N. dicksoni* cotyle of the cranium, again by using the manual template mesh deformation warping method. The left dentary was created by mirroring the reconstructed right dentary. These were positioned so that the condyles articulated with the cranium, the outer surfaces of the lower molars made contact with the inner surface of the upper molars, and the tips of the lower canines aligned with their ‘sockets’ in the cranium (see Figure 1).

It is important to note that the shape of the warp was determined by the existing regions of the *N. dicksoni* dentary; the need for the condyle to articulate with the cotyle to form the TMJ and for the coronoid process to fit between the cranium and the zygomatic arch. These requirements act as restraints on the warp such that the shape of the starting mesh (*D. maculatus* in this case) is not important in the sense that the warping process would always end with a similarly shaped posterior region of the mandible regardless of which taxon was used. We reiterate that *D. maculatus* was used because it was the most similar in shape [11] and therefore required less ‘warping’.

The *N. dicksoni* cranium was missing the following teeth: left I1-4, right I1, 3-4, both right and left C1 and right LDP2. The existing I2 and LDP2 on *N. dicksoni* were mirrored. All incisors were missing from the mandible. Incisors from *D. maculatus* were isolated, scaled and fitted into the empty tooth sockets on *N. dicksoni*. Figure 1 displays the completed reconstruction of *N. dicksoni*.

### Finite Element Models

The assembly of FEMs largely follows previously published procedures [30], [31], [35]. As the skull of *N. dicksoni* was not fully preserved, we were unable to assign multiple material properties to the digital reconstruction without introducing additional assumptions. Consequently, as in most FEA incorporating fossil material [30], [39], [67], all FEMs were homogeneous and assigned a single material property for cortical bone (*E* = 13.7 GPa, *v* = 0.3, where *E* is Young's modulus of elasticity and *v* is Poisson's ratio) [68] to enable direct comparisons between species. Poisson's ratio and Young's modulus are fundamental metrics in the comparison of stress or deformation for any material when strained elastically, including homogeneous materials [69]. Young's modulus is a measure of stiffness in the material, whereas Poisson's ratio is the negative ratio between transverse strain and longitudinal strain in an elastic material subjected to uniaxial stress [70].

Each homogeneous model was comprized of four-noded tetrahedral elements or ‘bricks’ (tet4). FEMs for QMF36357, AM1821, AM10756, UNSW Z20 and TMM M-6921 were comprized of 1564048, 1429714, 1799292, 1402103 and 1956942 bricks respectively. Tet4 models are theoretically less accurate than models comprized of tet10 elements. However, the models used in this study are large and any difference in accuracy between results from tet4 versus tet10 models will diminish as the number of elements is increased. Comparable analyses comparing tet4 and tet10 based models much smaller than those used here (<252000 elements) found differences of <10% [27].

### Modeling Masticatory Muscle Forces

Jaw elevators were modeled as seven muscle subdivisions: *temporalis superficialis*, *temporalis profundus*, *masseter superficialis*, *masseter profundus*, *zygomatico mandibularis*, *pterygoideus internus* and *pterygoideus externus* [32]. Proportions used for each jaw muscle division were based on muscle mass proportions from a dissected Virginia opossum (*Didelphis virginiana*) [71]. Muscle forces were predicted on the basis of maximum cross-sectional areas (CSA) using the ‘dry skull’ method [72]. To improve the accuracy of our CSA measurements, we used our FEMs to record the co-ordinates of ∼100 nodes at the perimeter of each muscle cross sectional area [73]. The FEM was moved to the correct orientation described by Thomason [70] to select nodes outlining the CSA. The node co-ordinates were then plotted in plane geometry software, GEUP 5 (version 5.0.3) and connected to form a multi-sided polygon. The area of the polygon was measured to estimate the CSA of each major jaw closing muscle. To minimize the incidence of artefacts at bite points and muscle origin and insertion areas, surface regions at these sites were tessellated using a network of stiff beam elements [74].

### Restraints, Loading Conditions and Scaling

Dasyurids frequently use a penetrating canine bite to kill prey [44], [75]–[78] which involves the application of a bending load [79]. We simulated bilateral canine biting (intrinsic load) and four extrinsic loads to simulate loads generated by struggling prey (axial twist, lateral shake, pullback and dorsoventral) for all models using protocols described by Attard et al. [35] and following McHenry et al. [31]. Extrinsic loads were modeled without applying bite forces so as to clearly reveal the different influences of each separate loading [31]. A gape angle of 35° was applied in all linear static load cases.

A considerable size range exists between specimens considered in the present study. The relationship between bite force and body mass is negatively allometric [26], [80]. To account for differences in body mass, a second series of load cases were solved following the scaling procedures of McHenry et al. [31]. Here, for each model, an estimate of bite force was made based on regression of body mass to predicted bite force for dasyuromorphians [*z* = 0.6998 (log *y*)+1.8735, where and *y* = mass (g) and *z* = bite force at canines (N)] [26], with body mass for each specimen predicted using the equation based on lower molar row length [log *y* = −1.075+3.209(log *x*), where *x* = lower molar length (mm), and *y* = mass (g)] as presented by Myers [81]. Muscle forces were then scaled for each specimen to achieve bite forces predicted on the basis of body mass. FEMs were solved using these scaled muscle forces. Prediction of bite force based on body mass using the regression equation provided in Wroe et al. [26] is close to that which would be expected following a 2/3 power relationship, whereby muscle force is proportional to area while body mass is proportional to volume [82]. The maximum bite force measured in Newtons (N) was also estimated for intrinsic loads (Table S2) using FEMs with un-scaled, specimen-specific estimated muscle forces (Table S3). Three dimensional approaches are likely to be more accurate than 2D based approaches [83].

A H-frame connecting the canines of the upper and lower jaws was used to apply extrinsic forces, with forces applied at the center of the frame [32], [35]. The force (N) applied to extrinsic loads was an arbitrary figure, applied for strictly comparative purposes, equivalent to 100 times the animal's estimated body mass for an axial twist, and 10 times the animal's estimated body mass for a lateral shake, pullback and dorsoventral shake [81]. Each simulation in which forces are applied with the anterior teeth (canines) restrained is a test for the hypothesis that stress will be highest for species with the longest rostrum.

**Figure 2:**
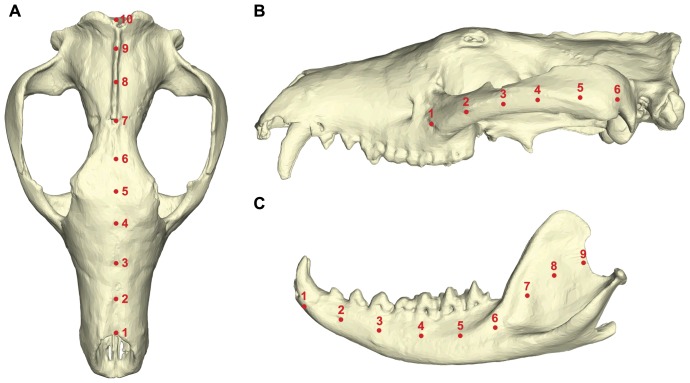
Position of nodes selected on each model to measure von Mises stress. Nodes were selected at equidistant points along the (A) mid-sagittal plane, (B) zygomatic arch and (C) mandible to measure the distribution of von Mises stress for each loading case.

Von Mises (VM) stress is a good predictor of failure in ductile materials such as bone [84], [85] and VM stress is used here as a metric for comparison between models following Attard et al. [35]. Nodes were selected at equidistant points along the mid-sagittal plane, zygomatic arch and mandible (Figure 2) following Attard et al. [35] and at each node values were calculated by averaging VM stress recorded in the surrounding elements to assess changes in stress magnitudes and distributions under different loadings.

Principal component analysis (PCA) was used to visualize differences between species in average VM stress values for equidistant nodes along the mid-sagittal plane (N = 10). PCA is an ordination technique that summarizes the maximum variation among a set of variables on few, uncorrelated axes (principal components) [86]. PCA was performed separately on VM stress values for each extrinsic load (axial twist, lateral shake, pullback and dorsoventral) and for a bilateral canine bite. All VM stress values were log transformed prior to PCA.

Differences in VM stress values between species were compared using a Kruskal-Wallis test, which is regarded as a multiple-group extension of the Man-Whitney test [87]. Significance values were corrected for multiple comparisons using Bonferroni corrections, as a conservative approach.

## Results

The predicted body mass (kg) of each species was generally within the expected range for each of the extant species (Table 1). Body mass estimates ranged from 0.78 kg for *D. hallucatus*, up to 32.49 kg for *T. cynocephalus*. However, the body mass estimated for *S. harrisii* of 14.20 kg was slightly above the upper limit observed for males (13 kg) [88], possibly because the teeth and skull are relatively large in this species. The robust craniodental morphology and relatively large teeth in *S. harrisii* are probably related to its habitual osteophagy, as has been observed in bone-cracking carnivorans [89]. To obtain body mass estimates for these taxa using simple or multiple regressions adjusted from cranidoental variables may lead to overestimates of body mass. Predicted maximum muscle forces for *N. dicksoni* (651 N) were relatively high, approaching those of the larger *S. harrisii* (685 N) (Table 1).

**Table 1:** Predicted body mass and masticatory muscle forces for modeled dasyuromorphians.

Species	Predicted body mass (kg)	Temporalis muscle force (N)	Masseteric muscle force (N)	Total muscle force (N)
*Dasyurus hallucatus*	0.78	67.60	55.89	123.49
*Dasyurus maculatus*	2.88	211.01	178.67	389.67
*Nimbacinus dicksoni*	5.25	282.38	368.33	650.71
*Sarcophilus harrisii*	14.20	300.46	384.73	685.19
*Thylacinus cynocephalus*	32.49	706.64	843.21	1,549.85

Predicted body mass (kg) calculated using the regression equation for dasyuromorphians provided by Myers [76] based on lower molar row length. Temporalis and masseteric muscle forces (N) were calculated based on cross-sectional area [67].

**Figure 3:**
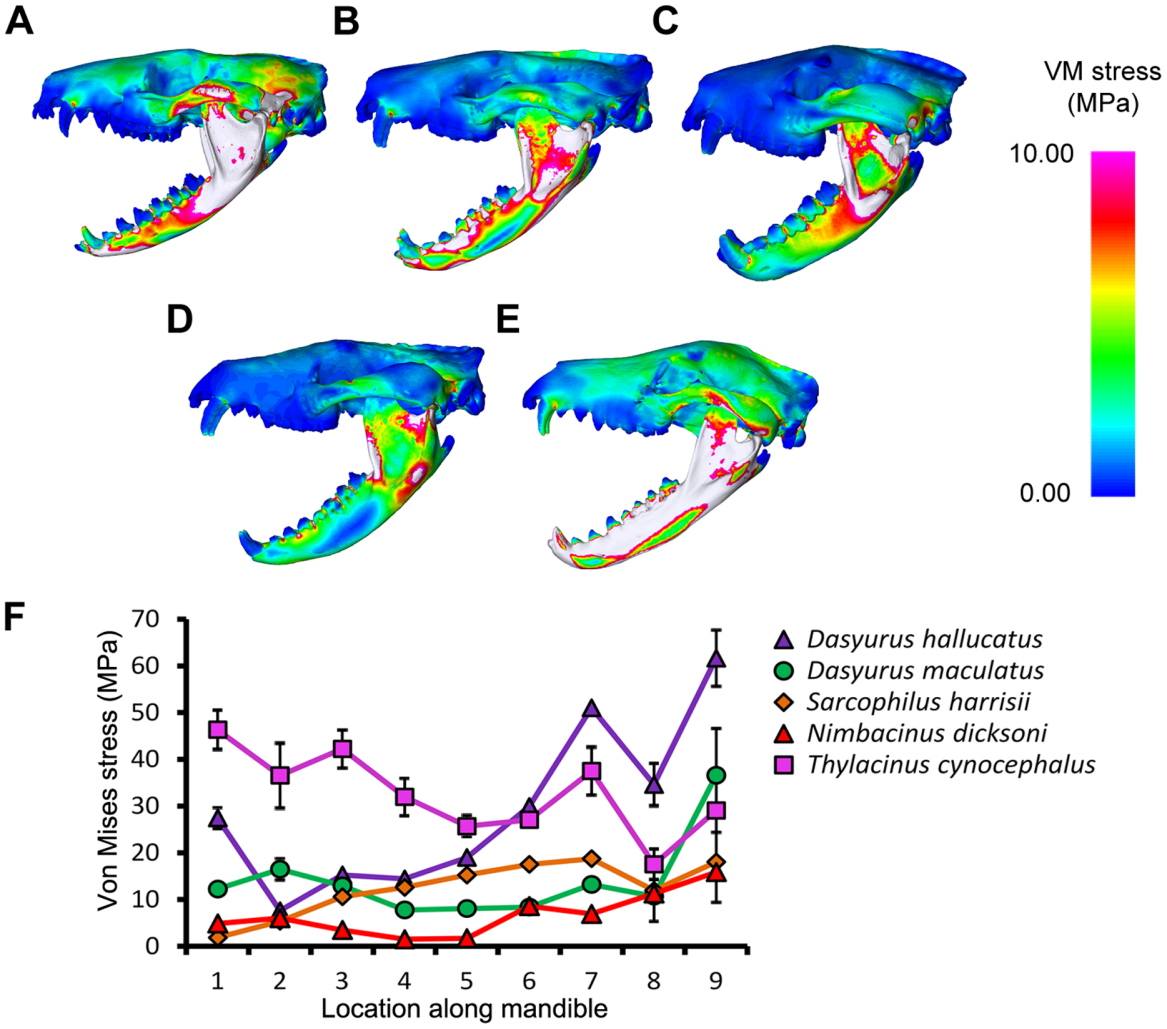
Von Mises stress under a bilateral canine bite in lateral view. The models are subjected to a load applied to both canines, with bite force scaled based on theoretical body mass. Species modeled were (A) *Dasyurus hallucatus*, (B) *Dasyurus maculatus*, (C) *Sarcophilus harrisii*, (D) *Nimbacinus dicksoni* and (E) *Thylacinus cynocephalus*. White colored regions of the skull represent VM stress above 10 MPa. (F) Distribution of von Mises stress was measured from anterior to posterior along the mandible.

**Figure 4:**
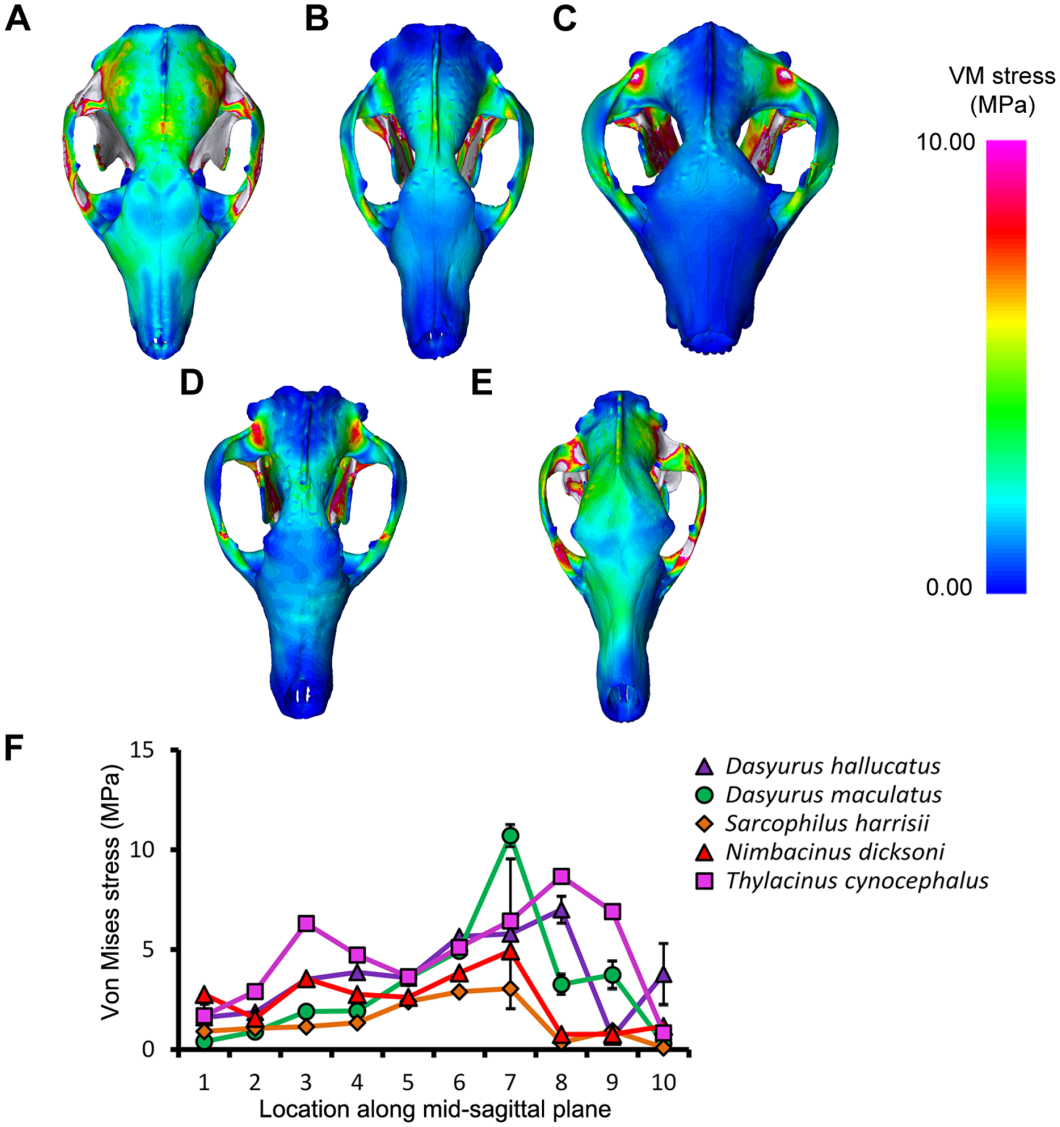
Von Mises stress under a bilateral canine bite in dorsal view. The models are subjected to a load applied to both canines, with bite force scaled based on theoretical body mass. Species modeled were (A) *Dasyurus hallucatus*, (B) *Dasyurus maculatus*, (C) *Sarcophilus harrisii*, (D) *Nimbacinus dicksoni* and (E) *Thylacinus cynocephalus*. White colored regions of the skull represent VM stress above 10 MPa. (F) Distribution of von Mises stress was measured from anterior to posterior along the mid-sagittal plane.

**Figure 5:**
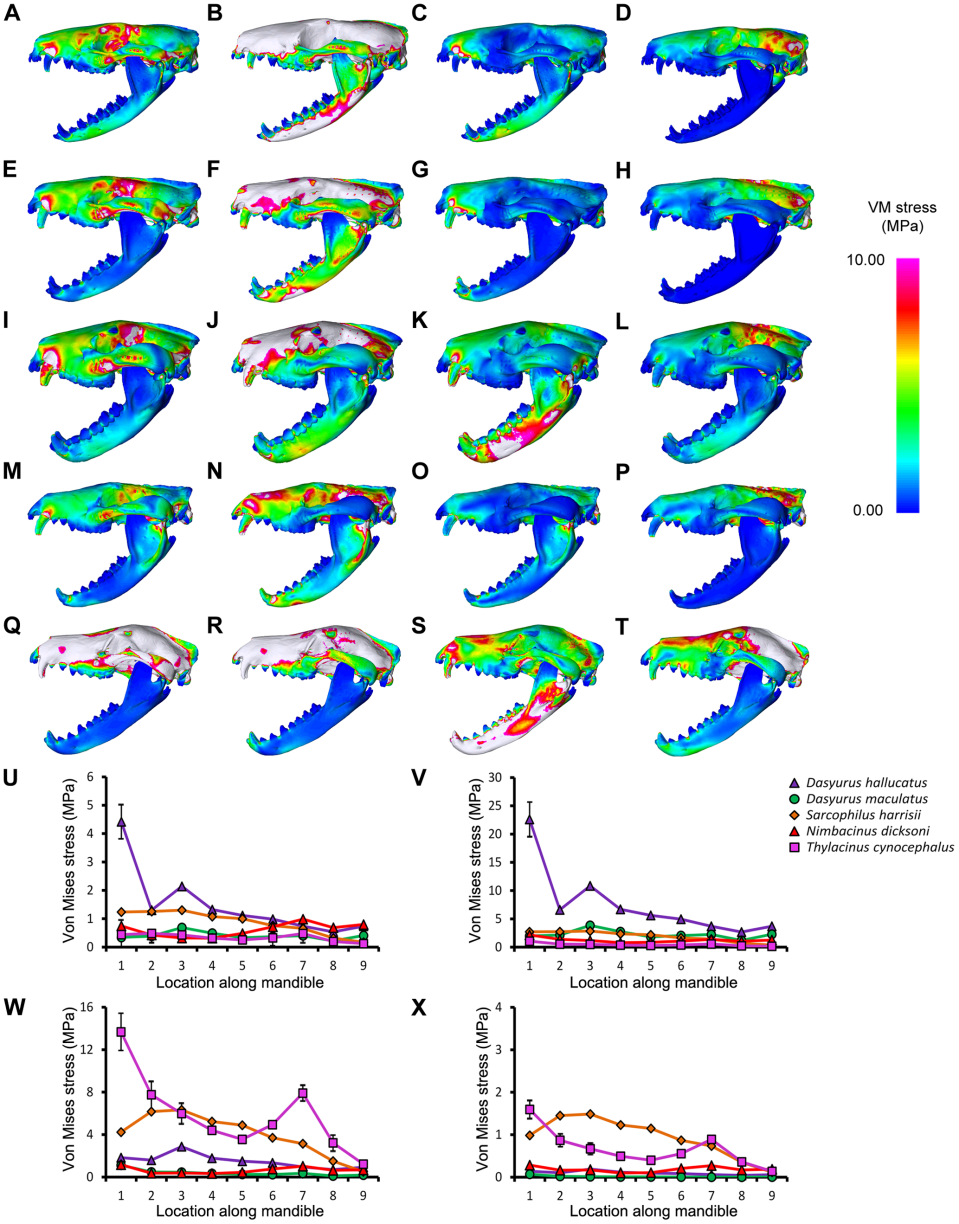
Von Mises stress under extrinsic loads in lateral view. The models are subjected to various loads applied to the canines, including a (A, E, I, M, Q) lateral shake, (B, F, J, N, R) axial twist, (C, G, K, O, S) pullback and (D, H, L, P, T) dorsoventral. The force applied was equivalent to 100 times the animal's estimated body mass for an axial twist, and 10 times the animal's estimated body mass for a lateral shake, pullback and dorsoventral shake. Species compared were (A–D) *Dasyurus hallucatus*, (E–H) *Dasyurus maculatus*, (I–L) *Sarcophilus harrisii*, (M–P) *Nimbacinus dicksoni* and (Q–T) *Thylacinus cynocephalus*. White colored regions of the skull represent VM stress above 10 MPa. Distribution of von Mises (VM) stress was measured from anterior to posterior along the mandible for a (U) lateral shake, (V) axial twist, (W) pullback and (X) dorsoventral.

**Figure 6:**
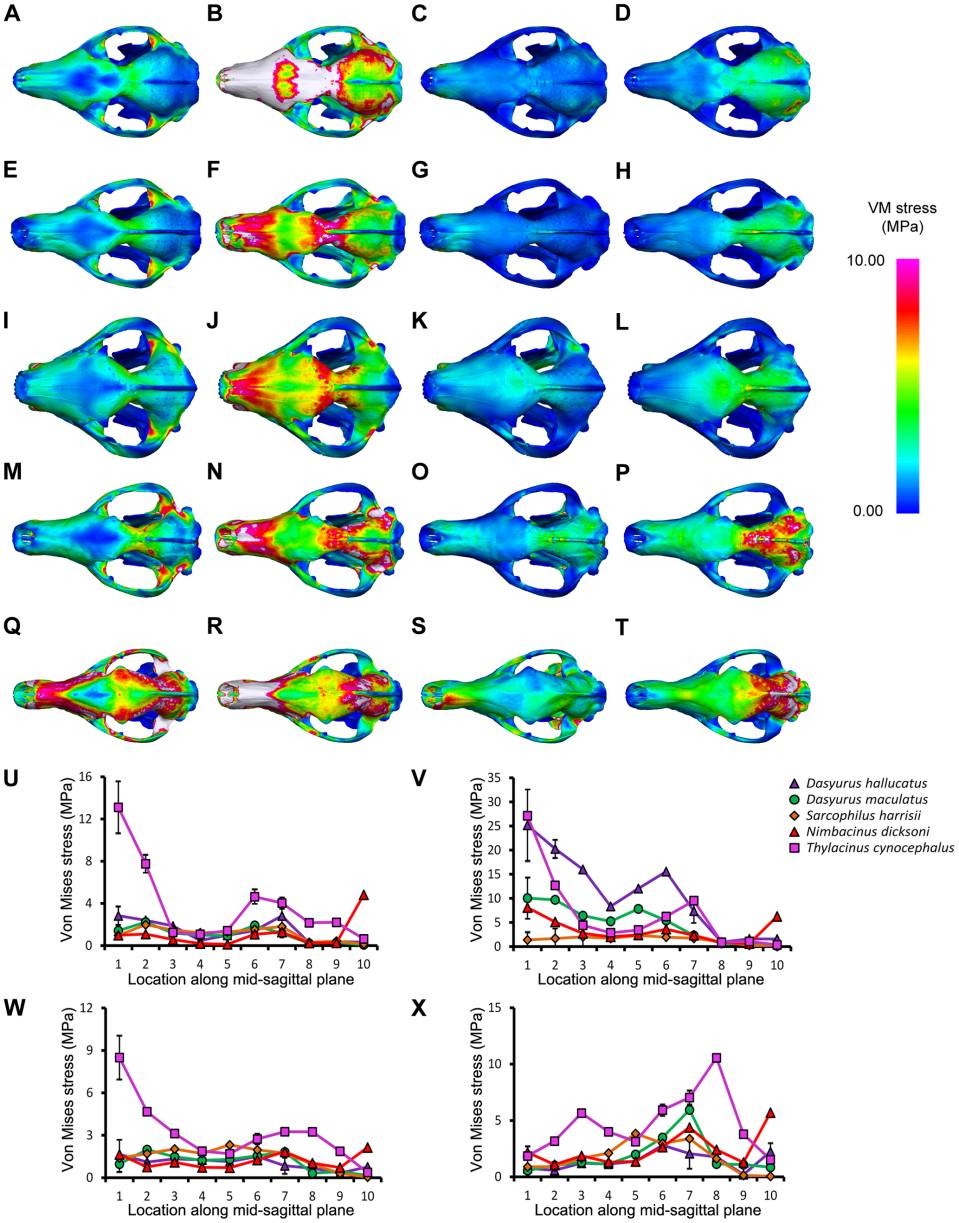
Von Mises stress under extrinsic loads in dorsal view. The models are subjected to various loads applied to the canines, including a (A, E, I, M, Q) lateral shake, (B, F, J, N, R) axial twist, (C, G, K, O, S) pullback and (D, H, L, P, T) dorsoventral. The force applied was equivalent to 100 times the animal's estimated body mass for an axial twist, and 10 times the animal's estimated body mass for a lateral shake, pullback and dorsoventral shake. Species compared were (A–D) *Dasyurus hallucatus*, (E–H) *Dasyurus maculatus*, (I–L) *Sarcophilus harrisii*, (M–P) *Nimbacinus dicksoni* and (Q–T) *Thylacinus cynocephalus*. White colored regions of the skull represent VM stress above 10 MPa. Distribution of von Mises (VM) stress was measured from anterior to posterior along the mid-sagittal plane for a (U) lateral shake, (V) axial twist, (W) pullback and (X) dorsoventral.

*Thylacinus cynocephalus* displayed comparatively high levels of VM stress in the cranium and mandible for most simulations (Figure 3–6, S3). This is consistent with results of Attard et.al. [35]. *Dasyurus hallucatus* showed relatively high levels of stress in the posterior of the mandible for a canine bite (Figure 3A), and along the ventral surface of the ramus for most extrinsic loads (Figure 5A–C).

The regions of highest stress along the dentary of *N. dicksoni* were located at the coronoid fossa and condylar process (Figure 3D). These regions of peak stress may be in part artifacts of reconstruction. Otherwise the dentary of *N. dicksoni* revealed similar stress patterns for a bilateral bite to *D. maculatus* (Figure 3). The distribution of stress for *N. dicksoni* in the cranium in response to a bilateral bite was intermediate between *S. harrisii* and *D. maculatus* (Figure 4). The magnitudes of stress along the mid-sagittal plane of *N. dicksoni* were slightly higher than for *S. harrisii* and lower than for *D. maculatus* (Figure 4F).

The highest stress in the cranium occurred at the zygomatic arch for all species in response to a bilateral canine bite (Figure 4). Von Mises stress along the zygomatic arch during a bilateral canine bite gradually increased posteriorly in *S. harrisii*, while stress peaked at node 3 in *T. cynocephalus* followed by a gradual decrease posteriorly (Figure S6A). The three other species displayed two peaks in stress along the zygomatic arch during a bilateral canine bite; one at the middle and the other at the posterior region of the zygomatic arch. *Thylacinus cynocephalus* was the only species to show two distinct peaks in stress for a bilateral bite along the mid-sagittal crest (Figure 4F). These stress points occurred at the temporal ridge and at the most narrowed region of the nasal (Figure 4E). Von Mises stress measured along the mid-sagittal crest for a bilateral bite revealed one point of peak stress halfway along the frontal of *D. maculatus*, *S. harrisii* and *N. dicksoni* and at the temporal ridge for *D. hallucatus* (Figure 4).

Stress was quite evenly distributed along the dentaries for all species in response to lateral shaking and axial twisting, with the exception of *D. hallucatus*, wherein stresses peaked anteriorly (Figure 5U–V) resulting in significantly higher VM stress values for that species compared to all others (χ^2^ = 32.87, *P*<0.0001). An axial twist resulted in much higher levels of stress along the mid-sagittal crest for *D. hallucatus* compared to all other species, and peaked at the anterior of the nasal and at the frontal (Figure 6B). *Sarcophilus harrisii* and *T. cynocephalus* showed higher levels of stress along the mandible for a pullback and dorsoventral shake than other species included in this study (Figure 5W–X). Comparisons of mandible VM stress values revealed significant differences between species after Bonferroni correction for both pullback (χ^2^ = 33.28, *P*<0.001) and dorsoventral shake (χ^2^ = 35.61, *P*<0.0001), with the exception of *T. cynocephalus* and *S. harrisii*. Two points of peak stress were apparent along the dentary for *T. cynocephalus* in these two simulations; one at the most anterior point, and the second at the coronoid fossa. Stress distribution along the dentary of *S. harrisii* followed a similar trend for a pullback and dorsoventral shake; peaking at the ramus inferior to M1 then gradually decreasing posteriorly.

**Figure 7:**
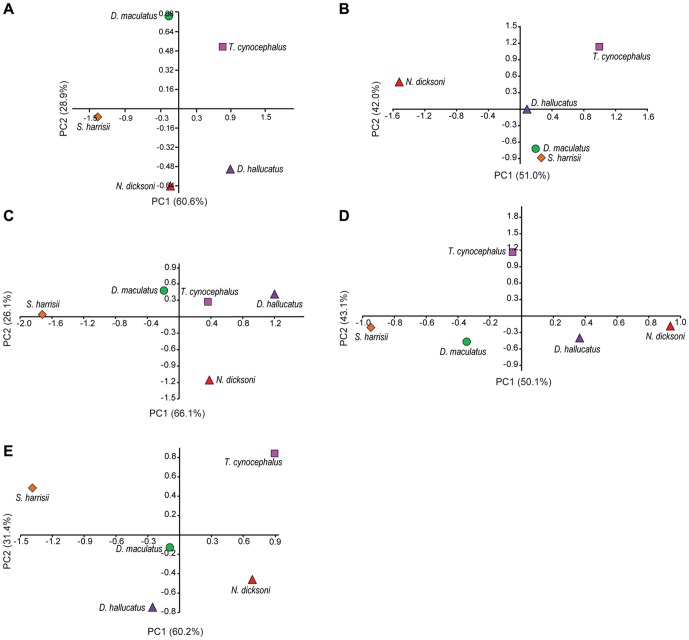
Principal components one and two of von Mises stress along mid-sagittal plane. Results of principal components analysis to compare stress among species along mid-sagittal nodes for each loading case, including (A) bilateral canine bite, (B) lateral shake, (C) axial twist, (D) pullback and (E) dorsoventral. Key to symbols: (pink square) *Thylacinus cynocephalus*; (red triangle) *Nimbicinus dicksoni*; (orange diamond) *Sarcophilus harrisii*; (green circle) *Dasyurus maculatus*; (purple triangle) *Dasyurus hallucatus*.

PCA results for mid-sagittal node VM stress values (Figure 7) showed that a high proportion of variance could be explained in all cases by two Principal Component (PC) axes (>85%). These plots provide an appreciation of interspecific differences across all 10 mid-sagittal nodes and bite simulations. PCA results indicate that the main axes of interspecific variance for all bites were explained by either nodes 1 and/or 7–10. *Thylacinus cynocephalus* and *S. harrisii* differed significantly for VM stress values under a bilateral bite at the canines (χ^2^ = 12.95, *P* = 0.04) and PCA results indicated separation of those two species along PC1 (60.6%), which largely explained variance in node 8 and node 10 (Figure 7A). PC1 for a bilateral canine bite revealed close similarities between *N. dicksoni* and *D. maculatus*, whereas PC2 (28.9%) clearly separated *N. dicksoni* from *D. maculatus* and reflected differences in node 7 (as seen in Figure 4).

For a lateral shake, PC1 (51.0%) explained change at nodes 1 and 10 and separated *T. cynocephalus* from *N. dicksoni* (Figure 7B), as also seen in Figure 6U. PC2 (42.0%) for a lateral shake revealed differences among all species for nodes 7 and 10, with *T. cynocephalus* closely resembling *N. dicksoni* compared to other species (Figure 7B). Interspecific differences were not significant for a lateral shake after Bonferroni correction, and before correction those distinguished *T. cynocephalus* from *N. dicksoni*, *D. maculatus* and *S. harrisii* (χ^2^ = 9.27, *P* = 0.01–0.03). PCA for an axial twist (Figure 7C) showed that the extremes of PC1 (66.1%) were delimited by *S. harrisii* and *D. hallucatus*, and the remaining taxa were located in between those two species. PC1 mainly accounted for differences among species for node 1 values (as seen also in Figure 6V), which were high for *D. hallucatus* and low for *S. harrisii*, whereas PC2 (26.1%) largely summarized node 10 values. Pairwise comparisons of VM stress values were not significant for an axial twist, however before Bonferroni correction, differences between *S. harrisii* and *D. hallucatus* (χ^2^ = 10.73, *P* = 0.03), *N. dicksoni* (χ^2^ = 10.73, *P* = 0.01) and *T. cynocephalus* (χ^2^ = 10.73, *P* = 0.04) could be distinguished from one another. For a pullback bite, PC1 (50.1%) mainly explained interspecific differences for node 10, and PC2 (43.1%) explained change at nodes 1 and 8 (Figure 7D). VM stress values for *T. cynocephalus* were different from those for *D. hallucatus* (χ^2^ = 14.37, *P* = 0.02) and *D. maculatus* (χ^2^ = 14.37, *P* = 0.04) for a pullback bite. For the dorsoventral shake, PC1 explained 60.2% of variance and reflected differences between *T. cynocephalus* and *S. harrisii*, whereas values across nodes were more similar among the remaining three species, located toward the middle of PC1 (Figure 7E). PC2 (31.4%) separated *D. hallucatus* from *T. cynocephalus*, and pairwise comparisons revealed node values to be different between those two species (χ^2^ = 14.78, *P* = 0.01).

## Discussion

Differences in biomechanical performance between the three extant dasyurids included in this study appear consistent with their respective known feeding behaviors. *Dasyurus hallucatus* showed comparatively higher levels of stress in most simulations than *S. harrisii* and *D. maculatus*. *Dasyurus hallucatus* eats invertebrates and other relatively small prey [60]–[62], which may not require adaptation to sustain the full range of extrinsic loads simulated here. This species shows particularly high VM stress in axial twisting, especially in contrast to *S. harrisii* However, it performs relatively well under pull-back loading, which may be linked to a capacity for pulling invertebrates from the ground. Observational studies on wild *D. hallucatus* will be required to confirm the functional role of their skull in prey acquisition. Future work on the comparative musculoskeletal anatomy and collection of in vivo or ex vivo biomechanical data of the extant species would likely improve the predictive power of current bite force and muscle force estimations. Overall consistencies found between known prey size and biomechanical performance for extant dasyuromorphians underscore the potential value of projections based on comparative FEA for extinct/fossil taxa.

Our comparative biomechanical modeling of dasyuromorphian skulls suggests considerable differences in predatory behaviors between the two thylacinids considered here. Our 3D based results indicate that the Oligocene to Miocene *N. dicksoni* had a high bite force for its size, comparable to that of extant dasyurids known to take relatively large prey, *D. maculatus* and *S. harrisii* [48], [60]. In light of similar levels of ‘carnassialization’ (development of relatively long, high amplitude vertical shearing crests) in the cheektooth dentition with *D. maculatus*, and a lack of obvious dental specialization consistent with regular bone-cracking, our results suggest a predominantly carnivorous diet for *N. dicksoni* that may have included relatively large prey. *Dasyurus maculatus* are opportunistic hunters, varying their diet in response to environmental disturbances and short-term fluctuations in prey abundance [54], [56]. They will prey on vertebrate species up to and sometimes exceeding their own body mass. Prey includes bandicoots, smaller dasyurids, possums, smaller macropodoids, snakes, lizards, birds and frogs, as well as invertebrates. Potential prey for a fox-sized thylacinid living in the closed forest communities of Riversleigh likely included many small to medium-size birds, frogs, lizards and snakes, as well as a wide range of marsupials, including bandicoots (peramelemorphians), dasyurids (dasyuromorphians), kangaroos (macropodoids), thingodontans (yalkiparidontians), marsupial moles (notoryctemorphians) and wombats (vombatoids) [12].

Although our FEA results for *N. dicksoni* suggest a capacity to kill prey approaching or exceeding its own body mass, its prey range may have been limited by competition with sympatric carnivores. The extent of niche overlap and competition within this ancient, medium-large sized carnivore community may have been partially alleviated by occupying different habitats and specializing in different hunting strategies. The recovery of a near complete skeleton of *N. dicksoni* [14] will provide further information on the locomotion and predatory behavior based on postcranial material; for example, was *N. dicksoni* as arboreal as the extant *D. maculatus*?

Differences in mechanical performance suggest that *T. cynocephalus* is unusual relative to other dasyuromorphians, including, *N. dicksoni*, as indicated by distinctly higher VM stresses than all other species in response to each loading case. *Thylacinus cynocephalus*, in contrast to *N. dicksoni*, has completely lost the metaconid on the lower molars and has a proportionately much larger postmetacrista on the upper molars. On the basis of traditional beam theory we predicted that taxa with longer rostra would exhibit higher stress [90], as evident in the long-snouted *T. cynocephalus* relative to shorter-snouted dasyuromorphians. Differences between *T. cynocephalus* and other species were also significant for three out of five simulations examined after conservative Bonferroni correction for multiple testing. These results further support the contention by Attard et al. [35] that niche breadth in *T. cynocephalus* may have been more limited and that it likely preyed on relatively small to medium-sized vertebrates such as wallabies, possums and bandicoots.

Although measures of skull performance in response to forces imposed by struggling prey revealed closer similarity between the fossil thylacinid *N. dicksoni* and large extant carnivorous dasyurids, than with *T. cynocephalus*, there were differences. Our reconstruction suggests that the TMJ was more elevated in *N. dicksoni* than in *D. maculatus*, and higher relative to the height of the cheektooth row. The TMJ is a complex joint and is important for occlusion and mastication [91], [92]. The position of the TMJ can influence bite strength and muscle activation [93]. The position of the TMJ along the anterior-posterior axis tends to lie closer to the plane of the tooth row in carnivorous taxa [11]. Conclusive determination of the precise position and morphology of the TMJ in *N. dicksoni* must await the discovery of more complete cranial material.

Morphological evidence from past studies further demonstrates diversity within this family. The smallest thylacinid, *Muribacinus gadiyuli*, is believed to have fed on relatively small vertebrates and invertebrates because it lacks a number of dental features present in large prey specialists (e.g., robust protoconids and brachycephalization) such as similarly sized *D. maculatus* [17]. The variety of feeding behaviors among thylacinids may have helped facilitate their co-existence within different ecological niches that were later filled by diversifying carnivorous dasyurids.

## Supporting Information

Figure S1 **Phylogenetic tree of dasyuromophians investigated in this study.** One of several recent assessments of the phylogenetic relationships of dasyuromorphians, including taxa that have been examined in this study (Wroe & Musser 2001).
(TIF)

Figure S2 **Interactive 3D pdf showing the digitally segmented cranium and mandible of** ***Thylacinus cynocephalus*** **.**
(PDF)

Figure S3 **Interactive 3D pdf showing the digitally segmented cranium and mandible of** ***Sarcophilus harrisii*** **.**
(PDF)

Figure S4 **Interactive 3D pdf showing the digitally segmented cranium and mandible of** ***Dasyurus maculatus*** **.**
(PDF)

Figure S5 **Interactive 3D pdf showing the digitally segmented cranium and mandible of** ***Dasyurus hallucatus*** **.**
(PDF)

Figure S6 **Von Mises stress along zygomatic arch for all loading cases.** Distribution of von Mises (VM) stress was measured from anterior to posterior along the zygomatic arch for a (A) bilateral canine bite, (B) lateral shake, (C) axial twist, (D) pullback and (E) dorsoventral.
(TIF)

Table S1 **Temporal and geographic distribution of thylacinid species.** Abbreviations: Aust, Australian mainland; E., Early; L., Late; M., Middle; Mio, Miocene; NG, New Guinea; NT, Northern Territory; Oligo, Oligocene; Plio, Pliocene; Qld, Queensland; Tas, Tasmania.
(PDF)

Table S2 **Maximum bite forces (N) for un-scaled homogeneous models for a bilateral canine bite.**
(PDF)

Table S3 **Muscle forces used for each jaw muscle division in un-scaled intrinsic models.** Species studied were *Dasyurus hallucatus*, *Dasyurus maculatus*, *Sarcophilus harrisii, Nimbacinus dicksoni* and *Thylacinus cynocephalus*. These were calculated using muscle mass proportions from dissected *Didelphis virginiana* (Turnbull 1970). Muscle forces were scaled for a bilateral canine bite by multiplying the muscle force by the ratio between bite force estimated using body mass regressions and maximum bite force estimated from the un-scaled model.
(PDF)

References S1 **Supporting Information references.**
(PDF)
