# Supplementary figures and images for: Virtual Reconstruction and Prey Size Preference in the Mid Cenozoic Thylacinid, Nimbacinus dicksoni (Thylacinidae, Marsupialia)
Source: PLoS One. 2014 Apr 9;9(4):e93088. doi: 10.1371/journal.pone.0093088 (PMC3981708; doi:10.1371/journal.pone.0093088)

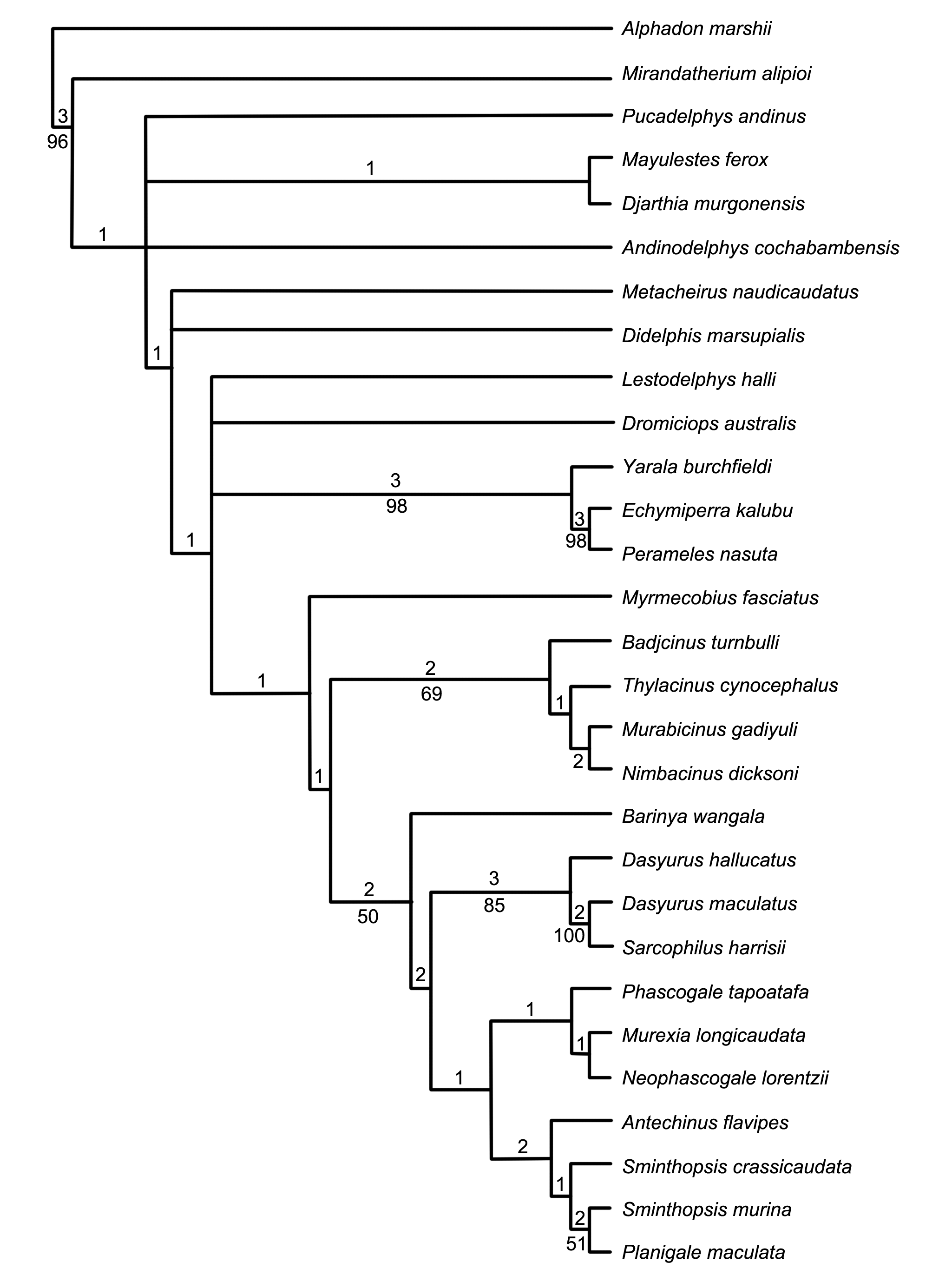

Supplement: Figure S1 — Phylogenetic tree of dasyuromophians investigated in this study. One of several recent assessments of the phylogenetic relationships of dasyuromorphians, including taxa that have been examined in this study (Wroe & Musser 2001). (TIF) [file pone.0093088.s001.tif]

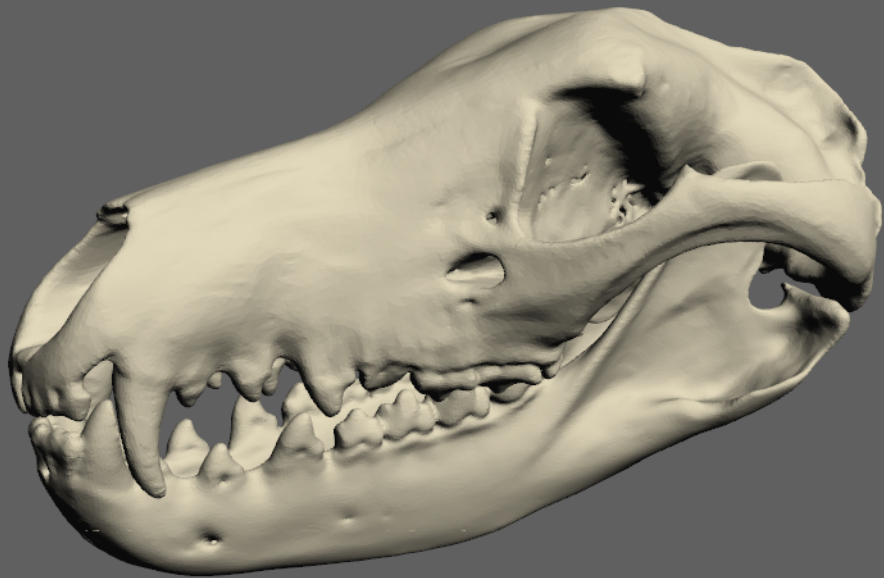

Supplement: Figure S2 — Interactive 3D pdf showing the digitally segmented cranium and mandible of Thylacinus cynocephalus . (PDF) [file pone.0093088.s002.pdf]

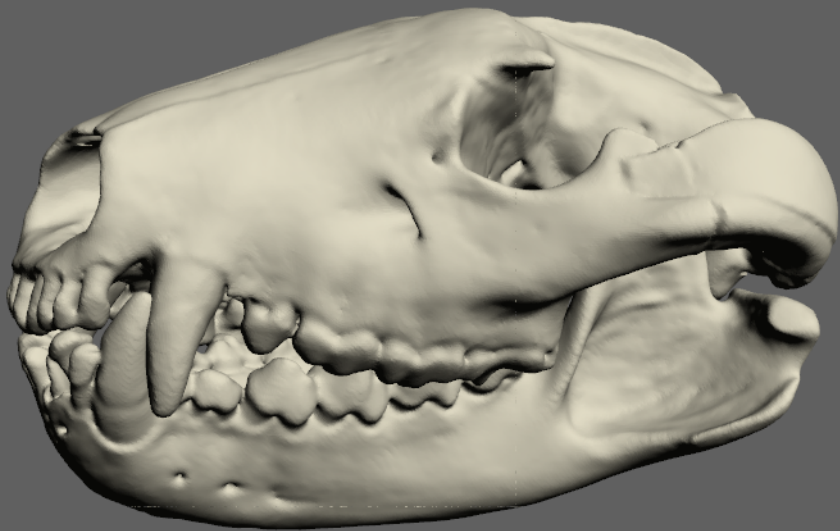

Supplement: Figure S3 — Interactive 3D pdf showing the digitally segmented cranium and mandible of Sarcophilus harrisii . (PDF) [file pone.0093088.s003.pdf]

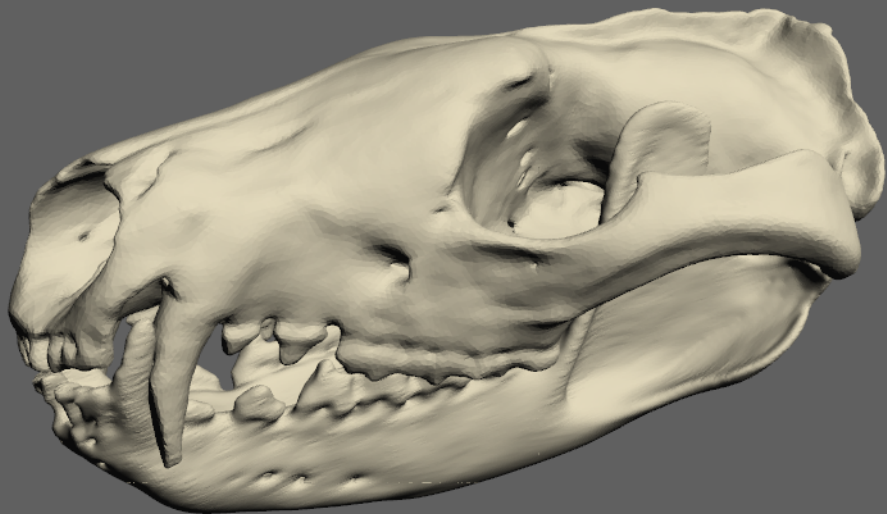

Supplement: Figure S4 — Interactive 3D pdf showing the digitally segmented cranium and mandible of Dasyurus maculatus . (PDF) [file pone.0093088.s004.pdf]

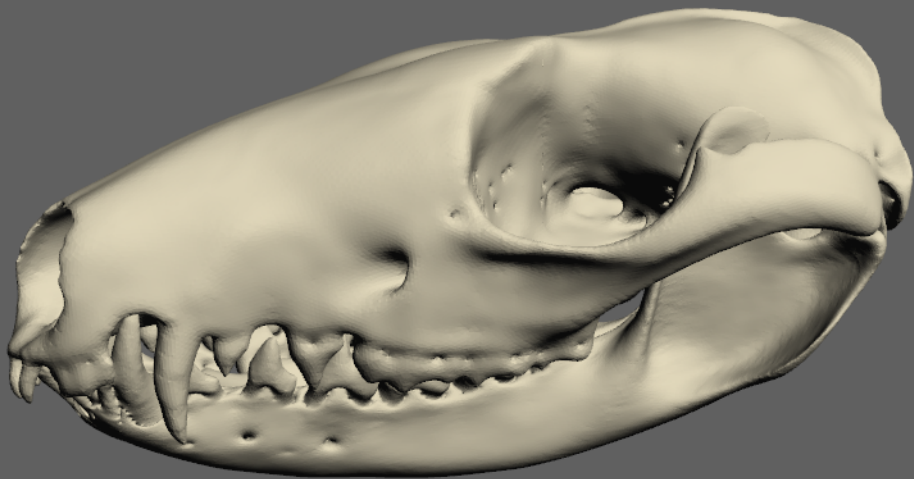

Supplement: Figure S5 — Interactive 3D pdf showing the digitally segmented cranium and mandible of Dasyurus hallucatus . (PDF) [file pone.0093088.s005.pdf]

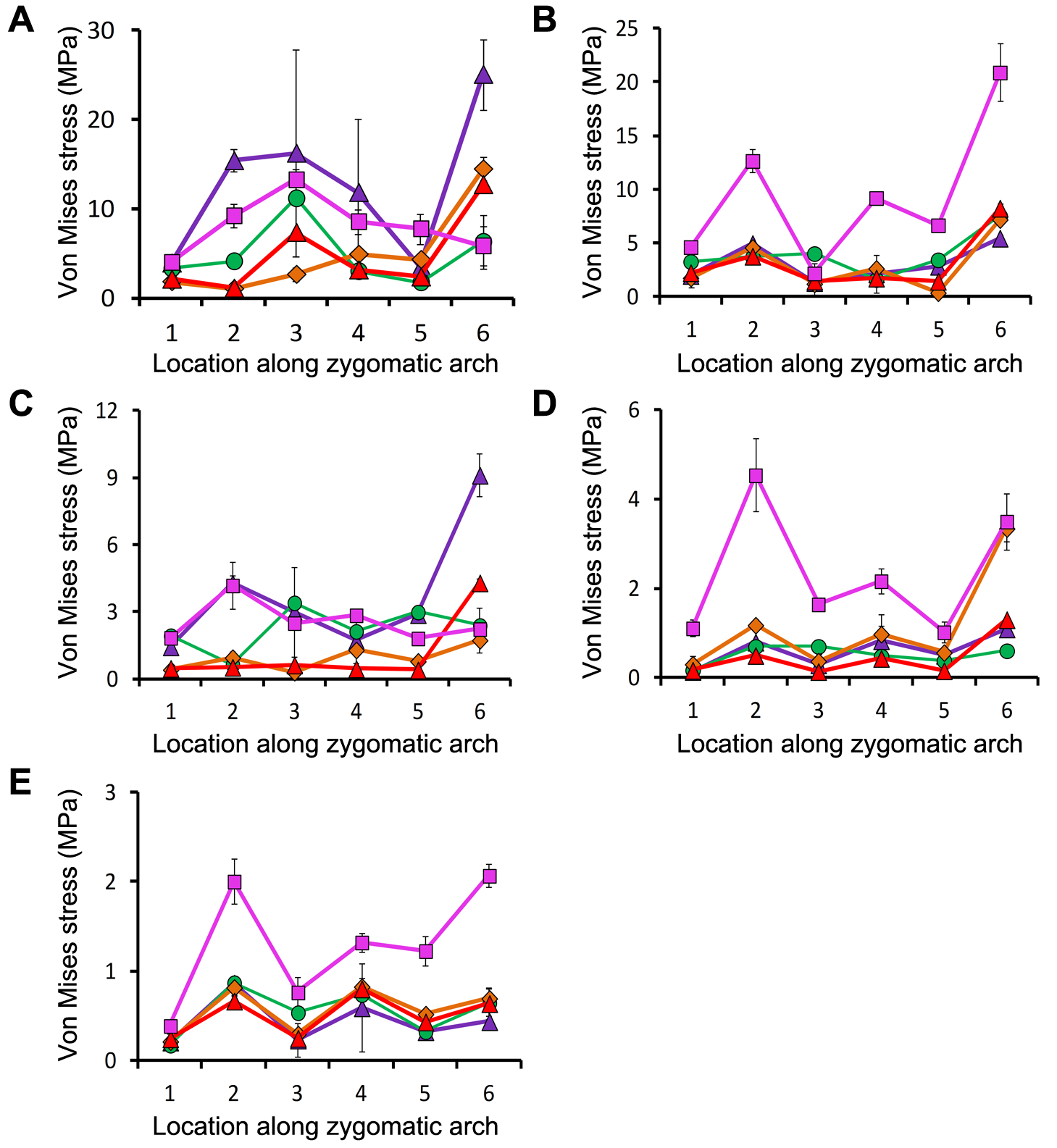

Supplement: Figure S6 — Von Mises stress along zygomatic arch for all loading cases. Distribution of von Mises (VM) stress was measured from anterior to posterior along the zygomatic arch for a (A) bilateral canine bite, (B) lateral shake, (C) axial twist, (D) pullback and (E) dorsoventral. (TIF) [file pone.0093088.s006.tif]
