# Supplementary material for: Virtual Reconstruction and Prey Size Preference in the Mid Cenozoic Thylacinid, Nimbacinus dicksoni (Thylacinidae, Marsupialia)
Source: PLoS One. 2014 Apr 9;9(4):e93088. doi: 10.1371/journal.pone.0093088 (PMC3981708; doi:10.1371/journal.pone.0093088)
Supplement: Table S1 — Temporal and geographic distribution of thylacinid species. Abbreviations: Aust, Australian mainland; E., Early; L., Late; M., Middle; Mio, Miocene; NG, New Guinea; NT, Northern Territory; Oligo, Oligocene; Plio, Pliocene; Qld, Queensland; Tas, Tasmania. (PDF) [file pone.0093088.s007.pdf]

Taxon	Age	Locality	Reference
Thylacinus cynocephalus	Plio-Recent	Aust, Tas, NG	Harris, 1808
Thylacinus potens	L.Mio	NT	Woodburne, 1967
Thylacinus megiriani	L.Mio	NT	Murray, 1997
Tjarrpecinus rothi	L.Mio	NT	Murray & Megirian, 2000
Muribacinus gadiyuli	M.Mio	Qld	Wroe, 1996
Mutpuracinus archibaldi	M.Mio	NT	Murray & Megirian, 2006
Maximucinus muirheadae	M.Mio	Qld	Wroe, 2001
Nimbacinus richi	M.Mio	NT	Murray & Megirian, 2000
Thylacinus macknessi	E.-M. Mio	Qld	Muirhead, 1992
Ngamalacinus timmulvaneyi	E.Mio	Qld	Muirhead, 1997
Wabulacinus ridei	E.Mio	Qld	Muirhead, 1997
Nimbacinus dicksoni	L.Oligo-M.Mio	Qld, NT	Muirhead & Archer, 1990
Badjcinus turnbulli	L.Oligo	Qld	Muirhead & Wroe, 1998
