# Supplementary material for: Virtual Reconstruction and Prey Size Preference in the Mid Cenozoic Thylacinid, Nimbacinus dicksoni (Thylacinidae, Marsupialia)
Source: PLoS One. 2014 Apr 9;9(4):e93088. doi: 10.1371/journal.pone.0093088 (PMC3981708; doi:10.1371/journal.pone.0093088)
Supplement: Table S2 — Maximum bite forces (N) for un-scaled homogeneous models for a bilateral canine bite. (PDF) [file pone.0093088.s008.pdf]

| Species | Maximum bite force (N) during a bilateral canine bite |
|---|---|
| *Dasyurus hallucatus* | 93.70 |
| *Dasyurus maculatus* | 299.81 |
| *Nimbacinus dicksoni* | 406.81 |
| *Sarcophilus harrisii* | 705.84 |
| *Thylacinus cynocephalus* | 835.94 |
