# Supplementary material for: Virtual Reconstruction and Prey Size Preference in the Mid Cenozoic Thylacinid, Nimbacinus dicksoni (Thylacinidae, Marsupialia)
Source: PLoS One. 2014 Apr 9;9(4):e93088. doi: 10.1371/journal.pone.0093088 (PMC3981708; doi:10.1371/journal.pone.0093088)
Supplement: Table S3 — Muscle forces used for each jaw muscle division in un-scaled intrinsic models. Species studied were Dasyurus hallucatus, Dasyurus maculatus, Sarcophilus harrisii, Nimbacinus dicksoni and Thylacinus cynocephalus. These were calculated using muscle mass proportions from dissected Didelphis virginiana (Turnbull 1970). Muscle forces were scaled for a bilateral canine bite by multiplying the muscle force by the ratio between bite force estimated using body mass regressions and maximum bite force estimated from the un-scaled model. (PDF) [file pone.0093088.s009.pdf]

	Muscle force distribution (in Newtons) of jaw muscle groups				
	D. hallucatus	*D. maculatus*	*N. dicksoni*	*S. harrisii*	*T. cynocephalus*
Temporalis superficialis	2.13	6.73	11.24	11.84	26.78
Temporalis profundus	2.13	6.71	11.21	11.80	26.70
Masseter superficialis	2.06	6.51	10.87	11.45	25.90
Masseter profundus	2.01	6.34	10.58	11.14	25.20
Zygomatico mandibularis	2.31	7.29	12.17	12.81	28.98
Pterygoideus internus	2.26	7.13	11.90	12.53	28.35
Pterygoideus externus	2.01	6.34	10.58	11.14	25.20
TOTAL	14.91	47.04	78.56	82.72	187.11
Scaled muscle force	0.67	0.52	0.59	0.68	1.02
