# Supplementary material for: Virtual Reconstruction and Prey Size Preference in the Mid Cenozoic Thylacinid, Nimbacinus dicksoni (Thylacinidae, Marsupialia)
Source: PLoS One. 2014 Apr 9;9(4):e93088. doi: 10.1371/journal.pone.0093088 (PMC3981708; doi:10.1371/journal.pone.0093088)
Supplement: References S1 — Supporting Information references. (PDF) [file pone.0093088.s010.pdf]

Harris GP (1880) Description of two new species of *Didelphis* from Van Diemen's Land. Trans Linn Soc Lond 9: 174-178.

Muirhead J (1992) A specialised thylacinid, *Thylacinus macknessi*,(Marsupialia: Thylacinidae) from Miocene deposits of Riversleigh, northwestern Queensland. Aust Mammal 15: 67-76.

Murray PF (1997) *Thylacinus megiriani*, a new species of thylacine (Marsupialia: Thylacinidae) from the Ongeva local fauna of central Australia. Rec S Aust Mus 30: 43-61.

Muirhead J, Archer M (1990) *Nimbacinus dicksoni*, a plesiomorphic thylacine (Marsupialia: Thylacinidae) from Tertiary deposits of Queensland and the Northern Territory. Mem Queensl Mus 28: 203-221.

Muirhead J, Wroe S (1998) A new genus and species, *Badjcinus turnbulli* (Thylacinidae: Marsupialia), from the late Oligocene of Riversleigh, northern Australia, and an investigation of thylacinid phylogeny. J Vert Paleontol 18: 612-626.

Murray PF, Megirian D (2000) Two new genera and three new species of Thylacinidae (Marsupialia) from the Miocene of the Northern Territory, Australia. The Beagle, Records of the Museums and Art Galleries of the Northern Territory 16: 145-162.

Murray P, Megirian D (2006) Cranial morphology of the Miocene thylacinid *Mutpuracinus archibaldi* (Thylacinidae, Marsupialia) and relationships within the Dasyuromorphia. Alcheringa 30: 229-276.

Turnbull WD (1970) Mammalian masticatory apparatus. Fieldiana: Geology 18: 149-356.

Woodburne MO (1967) The Alcoota Fauna, central Australia. An integrated palaeontological and geological study. Bureau of Mineral Resources Bulletin 87: 1-187.

Wroe S (1996) *Muribacinus gadiyuli*, (Thylacinidae: Marsupialia), a very plesiomorphic thylacinid from the Miocene of Riversleigh, northwestern Queensland, and the problem of paraphyly for the Dasyuridae. J Paleontol 70: 1032-1044.

Wroe S (2001) *Maximucinus muirheadae*, gen. et sp. nov. (Thylacinidae: Marsupialia), from the Miocene of Riversleigh, north-western Queensland, with estimates of body weights for fossil thylacinids. Aust J Zool 49: 603-614.

Wroe S, Musser A (2001) The skull of *Nimbacinus dicksoni* (Thylacinidae: Marsupialia). Aust J Zool 49: 487-514.
